# A Multi‐Center Clinical Trial to Evaluate the Efficacy of the Next Generation TriHex Technology Antiaging Regimen

**DOI:** 10.1111/jocd.70192

**Published:** 2025-04-25

**Authors:** Michael Gold, Charles Boyd, Deanne Mraz, Tiffany Robison, Faiza Shafiq, Alan D. Widgerow

**Affiliations:** ^1^ Tennessee Clinical Research Center Nashville Tennessee USA; ^2^ Boyd Birmingham Michigan USA; ^3^ DMR Research Westport Connecticut USA; ^4^ Alastin Skincare Inc., a Galderma Company Carlsbad USA; ^5^ Center for Tissue Engineering University of California, Irvine, Chief Scientific Officer Galderma Carlsbad California USA

**Keywords:** adipose tissue, aging skin, facial volume, skin elasticity, skin‐care products

## Abstract

**Background:**

Alastin Restorative Skin Complex Serum with TriHex Technology (Alastin Skincare Inc., Carlsbad, CA) has undergone reformulation to add Octapeptide‐45 and magnolol for improving facial skin hydration, elasticity, plumping, and overall skin quality. Detailed science on TriHex Technology 2.0 and magnolol has been recently published. This open‐label study was conducted to validate the science and demonstrate product efficacy and tolerability in individuals with moderate to severe facial skin aging.

**Methods:**

A multi‐center clinical study was conducted from February to August 2024. A total of 44 eligible participants (43 female, 1 male), ages 35–69 years, and Fitzpatrick skin Types I–VI were enrolled in and completed the study following 12 weeks of Alastin Restorative Skin Complex Serum 2.0 with TriHex Technology (RSC 2.0) use twice daily, along with an Alastin Skincare Inc. supportive regimen (cleanser, moisturizer, sunscreen used as needed). A 1‐week run‐in phase using the supportive regimen only was conducted, and eligibility was reassessed at baseline. Follow‐up visits were performed at Weeks 4, 8, and 12, where facial skin quality parameters were evaluated clinically and subject assessments and satisfaction questionnaires, biopsy collection, photography, and hydration and elasticity measurements were completed. Participants also maintained a study diary over the 12‐week treatment period.

**Results:**

Significant improvements for all clinically evaluated facial skin parameters were achieved at Week 12. Histology revealed increased stimulation of new adipocytes, epidermal keratinocyte HA (CD44), and new dermal collagen and elastin fibers post treatment.

**Conclusion:**

Alastin Restorative Skin Complex Serum 2.0 with TriHex Technology has been demonstrated to be safe and effective for improving overall facial skin quality, achieving notable improvements in volume, plumping, hydration, and extracellular matrix (ECM) collagen and elastin stimulation.

## 1 Introduction

Skin health and quality is a major focus in cosmetic dermatology, where improved options for restoring and maintaining skin health are highly sought after by patients. Humphrey et al. describe the impact that skin quality has on emotional health, quality of life, self‐perception, and interpersonal interactions [1]. One such option is the use of topical skincare products formulated with various actives that claim to target a variety of skin parameters, offering improvements over time. Alastin TriHex Technology is designed to recycle the extracellular matrix (ECM), increase collagen, elastin, and glycosaminoglycans, replacing the old waste products with new matrix components. Topical formulations with Alastin TriHex Technology have previously demonstrated increased collagen and elastin formation in the ECM on histological examination. Accordingly, Alastin Restorative Skin Complex Serum with TriHex Technology (Alastin Skincare Inc., Carlsbad, CA), formulated with a combination of peptides and other ingredients, has previously demonstrated efficacy for improving facial photoaging, fine lines and wrinkles, radiance, firmness, sagginess, and plumpness [2]. In an effort to expand the efficacy profile, Alastin Restorative Skin Complex Serum 2.0 with TriHex Technology has undergone a reformulation to include additional peptides and compounds for improving facial skin hydration, elasticity, plumping, and overall skin quality. This reformulation includes an added Octapeptide‐45, a proprietary peptide, which has been shown to promote the skin's production of its own high molecular weight Hyaluronic acid (HA) [3]. Importantly, the magnolol addition has the capacity to influence adipose tissue, creating a potential regenerative source that directly impacts skin health [4]. As such, Alastin Restorative Skin Complex Serum 2.0 with TriHex Technology—RSC 2.0 may provide additional beneficial effects for the improvement of overall facial skin quality, including volumization and plumping.

Facial skin quality assessments essentially measure various parameters seeking to approximate healthy, undamaged, and youthful‐appearing skin as far as possible. Assessing facial skin quality can be described using the four emergent perceptual categories (EPCs): facial skin tone evenness, skin surface evenness, skin firmness, and skin glow [5]. Collectively, these parameters cannot be measured or evaluated via single parameter assessments alone. Therefore, a multimodal approach has been undertaken in this study to assess facial skin quality through investigator assessments, subject assessments, questionnaires, bioinstrumentation, photography, skin topography analyses, and biopsy histology analyses. In addition, particular interest was focused on skin plumping and hydration (roughness) due to the unique constituents of the formulation, as alluded to above.

Here we present the effects of Alastin Restorative Skin Complex Serum 2.0 with TriHex Technology—RSC 2.0 on improving facial skin quality, volume, plumpness, hydration, and elasticity, along with the use of a daily facial skincare regimen over a 12‐week treatment period.

## 2 Material & Methods

This multi‐center prospective, open‐label study was approved by WCG IRB (Puyallup, WA). This study was conducted under applicable regulations in accordance with the principles of Good Clinical Practice. Study participants were consented prior to any study procedures. Eligible subjects were healthy adult men and women ages 35–70 years presenting with moderate to severe facial skin aging for a minimum of three parameters per the investigator clinical grading at screening and baseline and were Fitzpatrick skin types I–VI. Additionally, eligible subjects agreed to not use any new facial topical products, use only the study‐provided facial skin care regimen products, withhold all facial cosmetic procedures and treatments, maintain a stable weight, and minimize sun exposure for the duration of the study. Male subjects agreed to shave their face 12–24 h prior to all study visits. Individuals were excluded if they were nursing, pregnant, or planning to become pregnant, were current smokers or consumers of nicotine, had known allergies or sensitivities to any study product ingredients, had a current dermatologic disease of their facial skin (cancerous or pre‐cancerous lesions, any history of facial psoriasis, eczema, acne, rosacea, vitiligo, perioral dermatitis, herpes zoster/herpes simplex, or acanthosis, or any other inflammatory skin condition or active collagen disease, facial scars, excoriations or deformities), or a history of immunosuppression/immune deficiency disorders or severe elastosis, and/or excessive sun exposure, presence of facial piercings, beard, facial hair and/or tattoo(s), current use of oral or injectable medications known to cause weight loss or initiation of any new supplements within 14 days of screening, previous lifting threads, tissue grafting, or tissue augmentation with permanent implants, silicone or fat, or tattoo of the face, undergone treatment with chemotherapy or immunosuppressive agents within 6 months of screening, use of topical corticosteroids, topical prescription retinoids or over‐the‐counter retinoid‐containing products, cosmetic products with Alpha or Beta hydroxyl acids, Vitamin C, Hyaluronic acid, or other antiaging ingredients used on the face within 2 weeks prior to screening, or use of systemic retinoids within 3 months of screening, undergone facial treatments or procedures for resurfacing (laser, photo modulation, light, radiofrequency, ultrasound, chemical peel, dermabrasion, or other ablative/non‐ablative procedures), needling or mesotherapy, cryotherapy, neurotoxin injection, tissue‐augmenting therapy, contouring, or revitalization treatment of the face, injectable dermal fillers of the face, lipolytic injections (e.g., deoxycholic acid or other lipolytic substances), dental root canal, or sinus laser procedure within 6 months of screening. Additionally, individuals with a history of keloid formation, hypertrophic scars, other healing or bleeding disorders, or undergoing treatment with anticoagulants or inhibitors of platelet aggregation were excluded from biopsy participation.

Enrolled subjects completed up to five visits, including screening, baseline, and follow‐up visits at Weeks 4, 8, and 12. At the screening visit, eligible subjects were dispensed a cleanser (Ultra Calm Cleansing Cream, Alastin Skincare Inc., Carlsbad, CA), moisturizer (Ultra‐Light Moisturizer, Alastin Skincare Inc., Carlsbad, CA) and sunscreen (SilkSHIELD All Mineral Broad Spectrum Sunscreen SPF 30, Alastin Skincare Inc., Carlsbad, CA or HydraTint Pro Mineral Broad Spectrum Sunscreen SPF 36, Alastin Skincare Inc., Carlsbad, CA), as a supportive standardized facial skincare regimen. A 1‐week run‐in phase was then initiated using only the supportive skincare regimen products for twice‐daily use and sunscreen for use as needed. Following completion of the run‐in phase, participants that remained eligible to continue in the study at baseline per investigator assessments were dispensed the study topical Alastin Restorative Skin Complex Serum 2.0 with TriHex Technology (Alastin Skincare Inc., Carlsbad, CA) for facial skin application twice daily for 12 weeks. A study diary was also dispensed for subject documentation of any missed doses to ensure compliance. Incidence and severity of adverse events were assessed and collected at each visit.

### 2.1 Investigator Assessments

At each visit, investigators performed clinical grading of facial skin parameters (elasticity, wrinkles (global), skin surface roughness, pigmentation, erythema, pore size) using a Scientific Assessment Scale of Skin Quality (SASSQ) five‐point scale (0 = none, 1 = mild, 2 = moderate, 3 = severe, 4 = very severe) (pore size: 0 = fine, 1 = small, 2 = moderate, 3 = large, 4 = very large) and a Modified Griffith's 10‐point scale (0 = none, 1–3 = mild, 4–6 = moderate, 7–9 = severe) to assess facial skin fine lines/wrinkles (locations: forehead, Crow's feet, under eye, nasolabial, cheek lines, marionette lines), skin dullness (radiance), and loss of volume (plumpness).

### 2.2 Subject Assessments and Questionnaires

Subject Self‐Assessments were performed at baseline and after 4, 8, and 12 weeks of study test product use to self‐assess the severity of facial skin aesthetic parameters (dark spots/pigmentation, uneven skin tone (color), uneven skin texture (tactile), fine lines/wrinkles, skin redness) using a 10‐point scale (0 = none, 1–3 = mild, 4–6 = moderate, 7–9 = severe).

Subjects reported their perception and satisfaction with the study test product by completing a Test Product Experience Questionnaire for their facial skin at baseline (15 min post‐application) and at all follow‐up visits through Week 12 by selecting 1 of 5 responses (strongly agree, agree, neither agree nor disagree, disagree, strongly disagree) to the following statements: (1) overall, I am satisfied with the test product, (2) this product blends and absorbs well into my skin, (3) the product appears to improve my skin hydration, (4) my skin looks renewed and restored, (5) the product makes my skin more supple and plump, (6) the product soothes my skin, (7) I feel the product does not irritate my skin, (8) the product helps to improve the overall appearance of my skin, (9) the product makes my skin feel nourished, (10) my skin looks firmer, (11) use of this product makes me feel more confident in the appearance of my skin, (12) the product has improved my overall skin tone (color), (13) my skin appears more radiant, (14) my skin looks younger, rejuvenated, (15) I feel the product helps to improve my skin texture, (16) the appearance of my fine lines and wrinkles looks reduced, (17) make‐up applies well over the product (does not pill), (18) my skin looks lifted, (19) I notice a reduction in redness, (20) the product helps to minimize the appearance of pores, (21) my skin appears to glow, (22) I would continue using this product, (23) I would recommend this product to others.

Subject tolerability was assessed onsite at baseline, where participants completed a Subject Tolerability Assessment to grade the degree of redness, itching, burning, or stinging experienced 15 min post application of the study test product using a four‐point scale (0 = none, 1 = mild, 2 = moderate, 3 = severe). Participants with any irritation parameters scored a 3 (severe) in severity were withdrawn from the study, and an adverse event was captured.

### 2.3 Photography

Standardized facial imaging, including red and brown channel photography, was performed at each visit on clean skin, free of any study products, using a VISIA imaging system (Canfield Scientific Inc., Parsippany, NJ) at three clinical sites. Three different views were captured, including a frontal view and 45° angles on both the left and right sides of the face. A VISIA CR imaging booth with PRIMOS CR 300 (Phaseshift Rapid In vivo Measurement Of Skin) (Canfield Scientific Inc., Parsippany, NJ) was used to collect imaging at one clinical site, and 3D volume analysis of wrinkles was performed upon study completion. Cherry 3D facial imaging and topography analysis using Trace software (Cherry Imaging, Yokneam, Israel) was performed at another clinical site.

### 2.4 Bioinstrumentation Measurements

Facial skin elasticity changes were measured over the 12‐week treatment period from baseline pre‐application of the study test product and 30 min post application, and at all follow up visits using a Cortex Elasticity Probe (Cortex Technology, Aalborg, Denmark). Measurements were collected in triplicate by gently placing the device probe on the mid‐forehead, right and left cheekbone prominence, and in the same location at all visits to evaluate changes in facial skin retraction time.

Facial skin surface hydration levels were evaluated over the 12‐week treatment period from baseline pre‐application of the study test product and 30 min post application, and at all follow‐up visits on clean skin using a Corneometer CM 825 (Courage + Khazaka electronic GmbH, Köln, Germany). Measurements were collected in triplicate by gently placing the device probe on the forehead and each side of both cheeks and in the same location for all visits.

### 2.5 Biopsies

A total of 10 participants consented to having two 3 mm punch biopsies collected from a hair‐bearing region of the pre‐auricular area of the face at baseline, prior to product application, and at the final follow‐up visit post 12 weeks of study test product use. All biopsies were evaluated for histological changes pre (baseline) and post (12 weeks) study test product use by an independent dermatopathologist using the following histochemical and immunohistochemical stains: Movat, Herovici, H&E (Hematoxylin and Eosin), Perilipin‐1, and CD44. Biopsy image analysis was performed using ImageJ software [6] to quantify mean changes in Perilipin‐1 positive cells and CD44 expression, pre (baseline) and post (12 weeks) Test Product use.

Statistical analyses were performed by an independent statistician using descriptive statistics and paired sample *t*‐tests to compare changes from baseline to end of study (Week 12). *p* values < 0.05 were considered statistically significant. Subject questionnaires were tabulated, and the frequency and percentage of all responses were summarized for each question and timepoint. A higher percentage of favorable responses indicates positive subject perceptions of the study test product. All biopsy samples were analyzed by a blinded dermatopathologist for histological review, and select biopsy images were quantified via ImageJ software.

## 3 Results

### 3.1 Demographics

Overall, 44 subjects completed the study. Mean age was 52.2 years (Range: 35–69 years), and 97.7% (*n* = 43) were female and 2.3% (*n* = 1) were male. Study subject Fitzpatrick skin type distribution included, 4.5% (*n* = 2) were Type I, 43.2% (*n* = 19) were Type II, 34.1% (*n* = 15) were Type III, 11.4% (*n* = 5) were Type IV, 2.3% (*n* = 1) were Type V, and 4.5% (*n* = 2) were Type VI. Subject self‐perceived skin types included, normal (22.7%), dry (29.5%), oily (2.3%), combination (41.0%), and sensitive (4.5%).

### 3.2 Investigator Assessments

**FIGURE 1:**
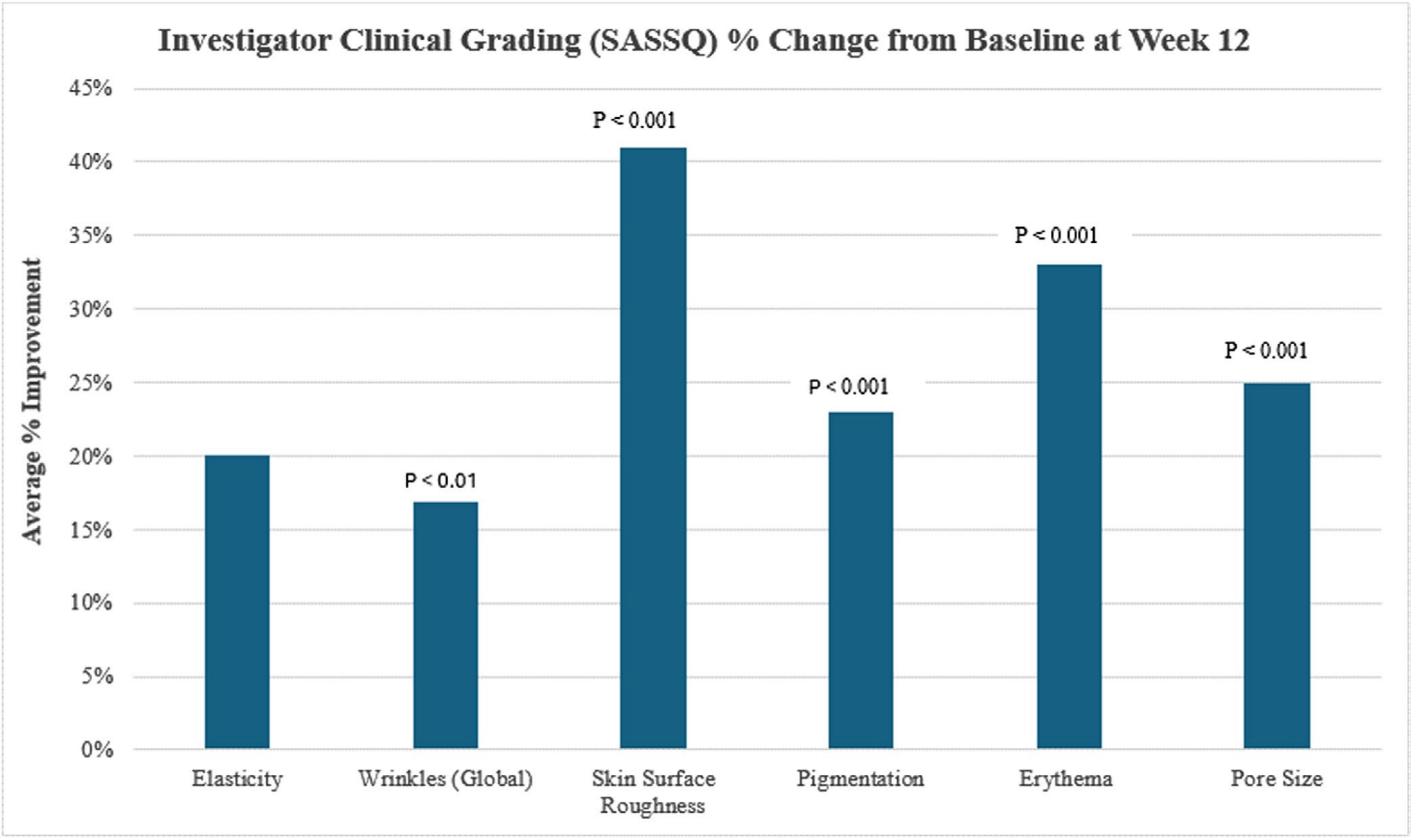
Investigator Clinical Grading using a SASSQ scale revealed significant improvements in overall skin quality collectively.

Statistically significant improvements were achieved across all clinical grading parameters evaluated from baseline at Week 12, where a mean improvement was demonstrated in elasticity by 20% (*p* < 0.001), wrinkle reduction by 17% (*p* < 0.01), skin surface roughness by 41% (*p* < 0.001), pigmentation by 23% (*p* < 0.001), erythema reduction by 33% (*p* < 0.001), and pore size reduction by 25% (*p* < 0.001) (Figure 1).

**FIGURE 2:**
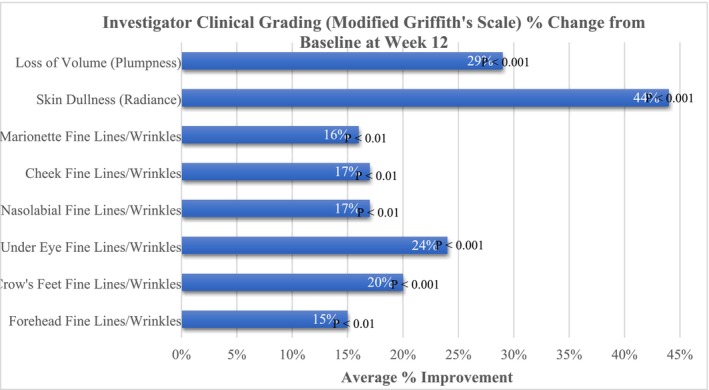
Investigator Clinical Grading using a Modified Griffith's scale demonstrated significant improvements in facial skin fine lines and wrinkles, radiance, and plumpness.

Further, targeted improvements were noted for facial skin fine lines/wrinkles, volume (plumpness), and dullness (radiance), where an average improvement of 15% (*p* < 0.01) was demonstrated for fine lines/wrinkles of the forehead, a 20% (*p* < 0.001) improvement in Crow's feet, a 24% (*p* < 0.001) improvement in under‐eye fine lines/wrinkles, a 17% (*p* < 0.01) improvement in both nasolabial and cheek lines, a 16% (*p* < 0.01) improvement in marionette lines, a 29% (*p* < 0.001) improvement in facial skin volume (plumpness) and a marked 44% (*p* < 0.001) improvement in facial skin dullness (radiance) (Figure 2).

### 3.3 Subject Assessments and Questionnaires

**FIGURE 3:**
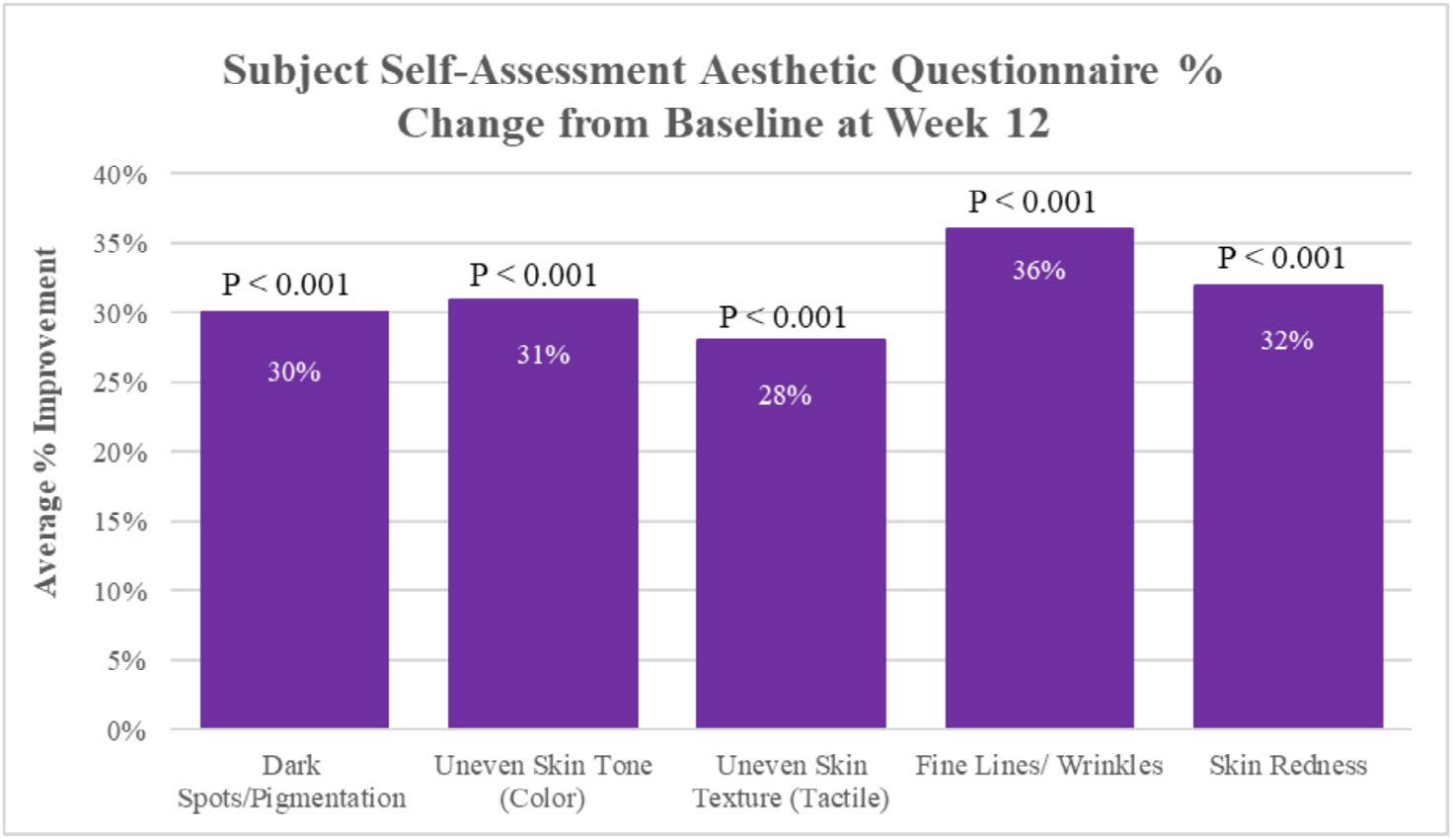
Subject Self‐Assessment performed pre and post 12 weeks of study test product use. Statistically significant improvements were achieved for all parameters evaluated at Week 12 compared to baseline.

After 12 weeks of study test product use compared to baseline, statistically significant improvements were demonstrated for all self‐assessed facial skin aesthetic parameters, where study participants reported a mean improvement of 30% (*p* < 0.001) in facial skin dark spots/pigmentation, 31% (*p* < 0.001) improvement in uneven skin tone (color), 28% (*p* < 0.001) improvement in uneven skin texture (tactile), 36% (*p* < 0.001) improvement in fine lines/wrinkles, and 32% (*p* < 0.001) improvement in facial skin redness (Figure 3).

At both the Weeks 8 and 12 follow‐up visits, 100% of study participants agreed that the study test product did not irritate their skin; 97.70% of participants agreed that the test product blends and absorbs well, and 93.20% of participants reported overall satisfaction with the study test product at the Weeks 8 and 12 follow‐up visits, as well. Additionally, after 12 weeks of study test product use, 97.70% agreed they would continue using the study test product and that the product appeared to improve skin hydration; 95.50% agreed that they would recommend the study test product to others; 93.20% agreed that the test product made their skin feel nourished; 90.90% agreed the test product helped improve their skin texture and that make‐up applied well over the product (does not pil); 88.60% agreed the test product improved the overall appearance of their skin; 86.40% agreed the study test product made their skin look more supple and plump, soothed their skin, and that their skin appeared to glow; 84.10% agreed their skin looked renewed and restored; 81.80% agreed product use made them feel more confident in the appearance of their skin and that the test product improved their overall skin tone (color); 79.50% agreed their skin appeared more radiant; 70.50% agreed that their skin looks firmer and that they noticed a reduction in redness; 68.20% agreed their skin looks lifted, the appearance of fine lines and wrinkles looks reduced, and that the product helped minimize the appearance of pores; and 63.60% agreed their skin looks younger and rejuvenated.

### 3.4 Photography

**FIGURE 4:**
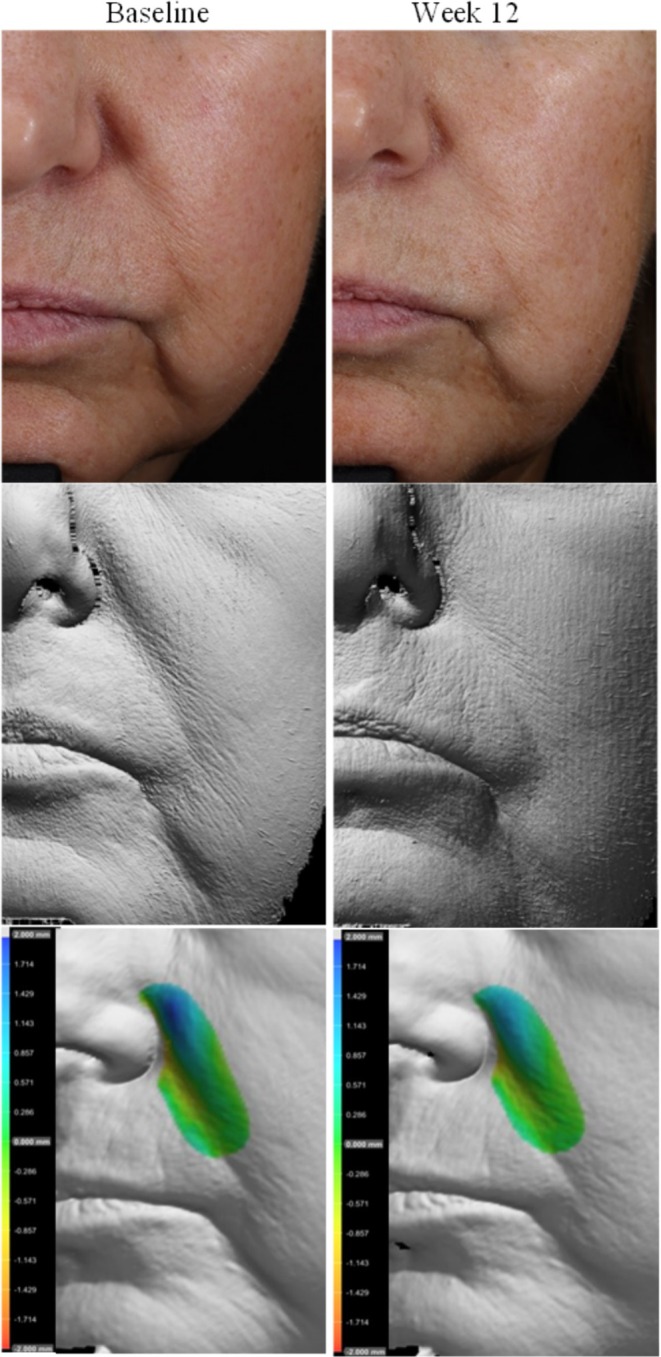
Female, age 62 years, Non‐Hispanic or Latino, Fitzpatrick skin Type II. Standard imaging (top), clay model (middle), volume analysis (bottom), pre (left) and post 12 weeks (right) of study test product use.

**FIGURE 5:**
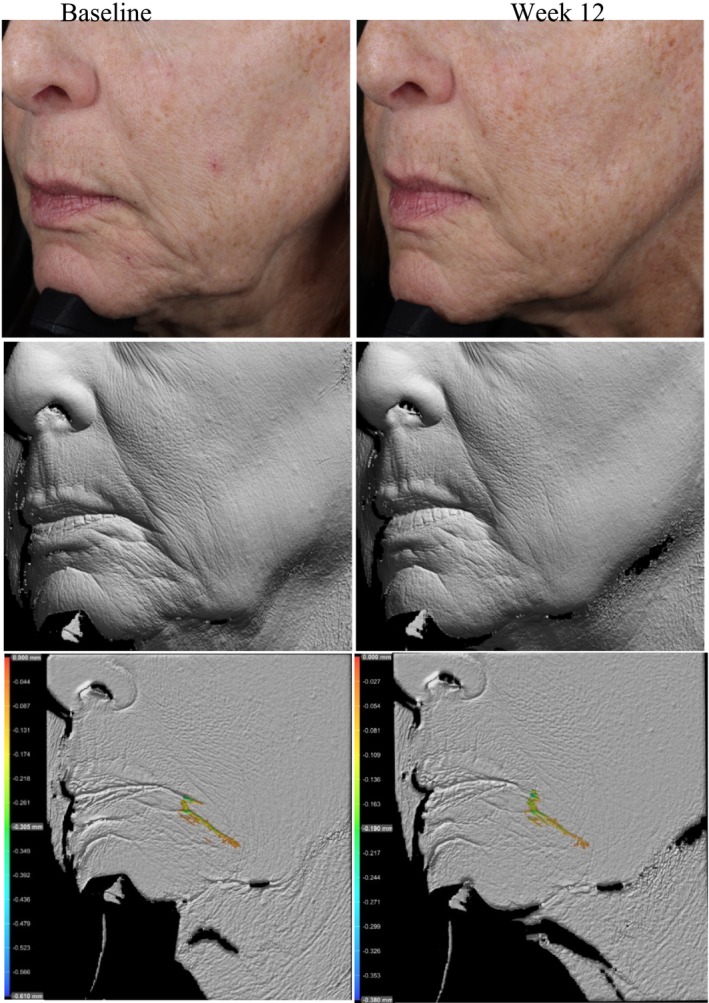
Female, age 60 years, Non‐Hispanic or Latino, Fitzpatrick skin Type II. Standard imaging (top), clay model (middle), volume analysis (bottom), pre (left) and post (right) 12 weeks of study test product use.

**FIGURE 6:**
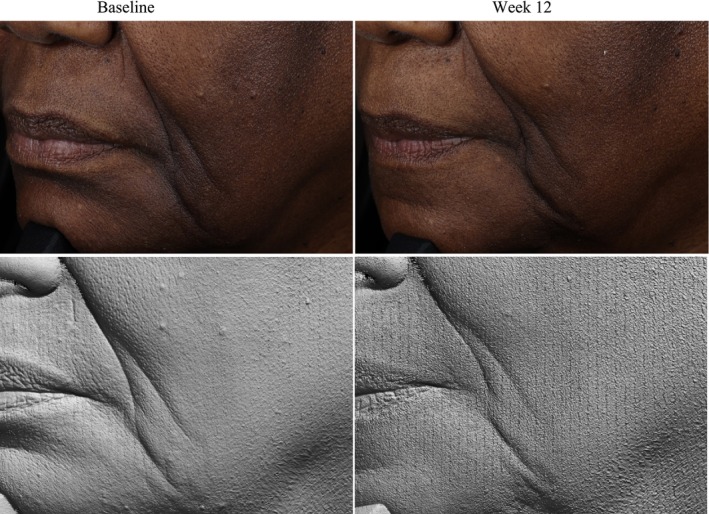
Female, age 65 years, Non‐Hispanic or Latino, Fitzpatrick skin Type VI. Standard imaging (top), clay model (bottom), pre (left) and post (right) 12 weeks of study test product use.

Photography and volume analysis reflected similar improvements as demonstrated in the clinical grading scores for wrinkle volume reduction and skin texture enhancement across multiple facial areas where 100% of patients analyzed showed significant volume improvements (Figures 4, 5, 6). An average wrinkle volume improvement of 28.13% was achieved for marionette lines (33.66%) (*p* < 0.001), Crow's feet (27.95%) (*p* < 0.001), glabellar (28.07%) (*p* < 0.001) and nasolabial wrinkles (17.88%) (*p* < 0.01) after the 12‐week treatment period. Wrinkles roughness (smoothness) improvements were also noted, where 100% participants achieved an average improvement of 20.15% for marionette lines (33.80%) (*p* < 0.001), Crow's feet (12.14%) (*p* < 0.01), and glabellar wrinkles (24.91%) (*p* < 0.001).

Additional wrinkle volume analysis of the cheek (both right and left) and marionette/nasolabial areas revealed moderate average improvements in wrinkle volume for both facial regions (cheeks: 0.571 cc, marionette/nasolabial areas: 0.517 cc), with a total cumulative improvement of 5.14 cc in the cheek areas and 8.8 cc in marionette/nasolabial areas across all participants analyzed post 12 weeks of study test product usage.

### 3.5 Bioinstrumentation

#### 3.5.1 Elasticity and Hydration Analysis

**FIGURE 7:**
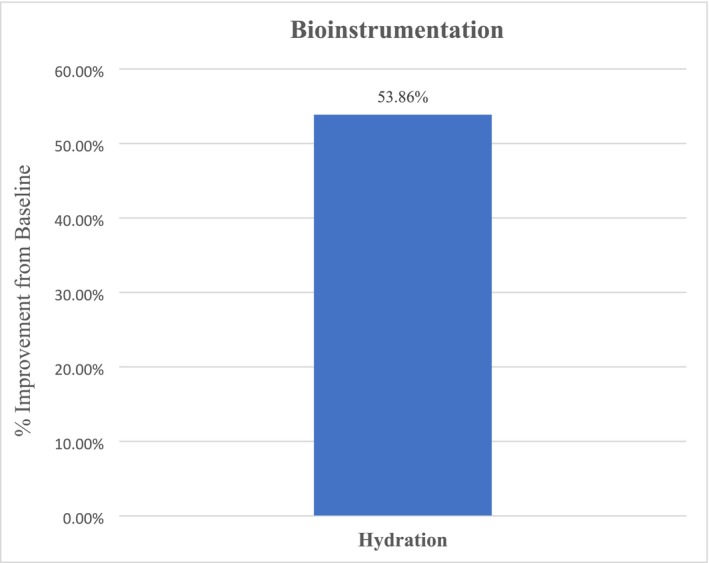
An overall increase of 53.86% (*p* < 0.001) in facial skin hydration was achieved post 12 weeks of study test product usage.

Overall, facial skin elasticity analysis revealed an average improvement of 4.55% for all facial areas (right, left, center), although not statistically significant (*p* = 0.47) at Week 12 from baseline. Corneometer facial skin hydration analysis revealed an overall statistically significant and notable increase in hydration of 53.86% (*p* < 0.001) for all facial areas (right, left, center) after 12 weeks of study test product use from baseline (Figure 7).

#### 3.5.2 Biopsies

**FIGURE 8:**
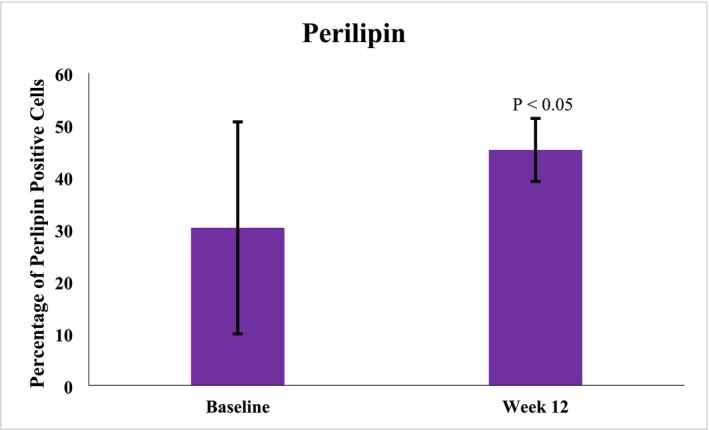
Percentage of Perilipin‐1 positive cells pre (baseline) and post (12 weeks) study test product use, where a 39.6388% increase was achieved at Week 12.

**FIGURE 9:**
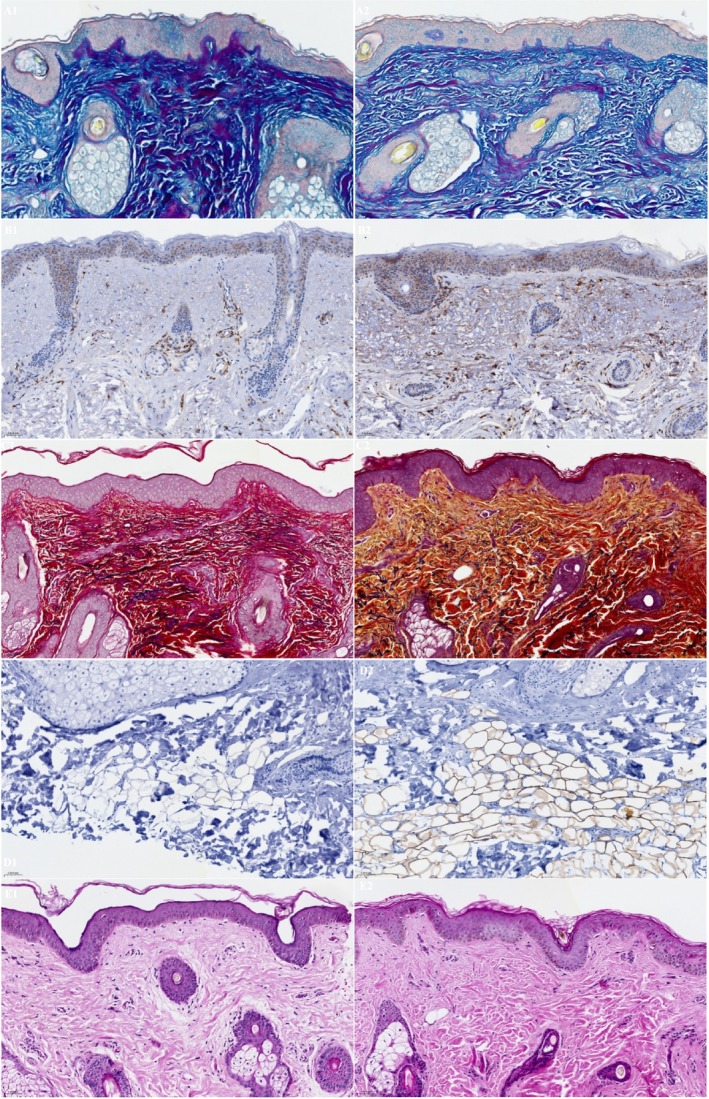
Study participant biopsy stains (400 × magnification) pre (left; A1–E1) and post (right; A2–E2) 12 weeks of study test product usage. (A, Herovici stain) female, age 44, Fitzpatrick Type II. (B, CD44 stain) female, age 61, Fitzpatrick Type I. (C, Movat stain) female, age 35, Fitzpatrick Type IV. (D, Perilipin‐1 stain) female, age 44, Fitzpatrick Type II. (E, H&E stain) female, age 35, Fitzpatrick Type IV.

A total of 10 subjects, female (*n* = 9) and male (*n* = 1), ages 35–61 years (mean age 47.6 years), Fitzpatrick skin Types I (*n* = 1), II (*n* = 3), III (*n* = 2), IV (*n* = 2), and VI (*n* = 2) elected to have 3 mm punch biopsies collected from a hair‐bearing region of the preauricular area, pre‐application of study test product usage at baseline and after 12 weeks of twice daily use, including the preauricular areas. Biopsy image analysis revealed statistically significant average increases in Perilipin‐1 positive cells after 12 weeks (45.18659%) of study test product usage from baseline (30.24%) (Figure 8). Additional Image J analysis demonstrated significant average increases in CD44 expression at Week 12 (0.05126832 AU) (*p* < 0.001) from baseline (0.02582526 AU), 98.52% respectively. Figure 9 demonstrates histology photomicrographs at Week 12 (right; A2–E2) compared to baseline (left; A1–E1). Herovici staining of baseline biopsy sections (A1) is consistent with mature collagen (stained magenta) and a baseline ratio of mature to immature collagen of approximately 4:1. This is compared to the Week 12 (A2) histology showing a marked increase in new (immature) collagen fibers within the reticular dermis, as demonstrated by the light blue staining, and with a 1:4 ratio of mature to immature collagen at Week 12. CD44 immunohistochemical staining at baseline demonstrates partial staining of a minority of epidermal keratinocytes (B1). At Week 12, complete circumferential staining of nearly all of the keratinocytes in the epidermis is noted, with expression within some of the dermal fibroblasts as well (B2). Movat staining at baseline histology sections demonstrates relatively short and somewhat fragmented elastic fibers within the dermis, with vertically oriented elastic fibers within the papillary dermis that are sparse (C1). At Week 12, Movat stained sections demonstrate longer elastic fibers as compared to baseline and increased fine, vertically oriented elastic fibers within the superficial and papillary dermis (C2). The histology section at baseline (D1) did not demonstrate any positive adipocytes with Perilipin‐1 immunohistochemical staining. Week 12 histology (D2) revealed increased expression and complete circumferential staining with Perilipin‐1 positive adipocytes in the vast majority of adipocytes. This expression is consistent with healthy intact adipocytes. H&E staining at baseline revealed solar elastosis within the superficial dermis, where the overlying epidermis is somewhat effaced with flattening of rete peg architecture, and relatively thin collagen fibers with increased space between the fibers (E1). In contrast, the post‐treatment Week 12 H&E‐stained sections show no solar elastosis, with epidermal rete peg architecture restoration, and dermal collagen fibers that appear thicker and more compact (E2).

#### 3.5.3 Tolerability and Safety

Tolerability assessments evaluated at baseline following the first application of the study test product demonstrated mild redness in 13.6% of study participants, mild burning (4.5%), mild stinging (2.27%), and itching (0%).

Two possibly related adverse events of irritant dermatitis (1 mild, 1 moderate) of the face were reported at one clinical site, both of which were self‐limiting and transient in nature, not affecting subject participation.

## 4 Discussion

Facial aging takes on various forms related to extrinsic and intrinsic stressors. These include the appearance of fine lines, loss of radiance, glow, and elasticity, and a perceived facial atrophy that contributes to these changes in appearance. This atrophic appearance appears to be related to ECM structural support changes to a certain extent, but a major component of the atrophy relates to changes within the fat compartment [7]. This includes changes to the dermal white adipose tissue, as well as to the subcutaneous fat tissue where photodamage initiates fibrotic changes within these compartments, resulting in decreased facial volume and important adipokine reduction that has a major impact on skin health [7]. In an effort to limit these changes and reconstitute some of this regenerative fat, an advanced formulation has been developed building on the positive results experienced over the years of the first rendition of Alastin Restorative Skin Complex Serum with TriHex Technology. These include ECM modulation with removal of damaged components and replacement with new collagen and elastin, and a certain amount of fat tissue stimulation. The new formulation, with the addition of Octapeptide‐45, has been validated to improve elastin levels, stimulate hyaluronic acid, improve basement membrane constitution, and impact fibroblast senescence [3, 8]. In addition, the inclusion of magnolol has created a targeted focus for fat tissue stimulation encouraging the conversion of preadipocytes to healthy new adipocytes, promoting fat tissue reconstitution [4]. Cumulatively, the constituents and the anticipated effects on different parameters of skin health have been demonstrated as effective in this study of 44 participants across multiple skin types.

Investigator assessments showed statistically significant improvements in all parameters measured, with marked improvements demonstrated in facial skin roughness, radiance, and plumpness. This is particularly important as the improved fat component and adipokine secretion, and increased HA levels, as evidenced by increased Perilipin‐1 and increased CD44 levels, would be expected to create this improved plumpness and skin health. Patient assessments bore out the improvements in general appearance (fine lines, redness, tone, pigmentation) and they reported ease of use, elegant formulation, and improvement with continued use of the product. Objective criteria for these observed parameter improvements were represented by photographic and volume analyses, hydration, and elasticity measurements, again bearing out the clinical and patient observations.

A total of 10 participants underwent biopsies. The first area for examination was the extracellular matrix (ECM) noting changes in the structural proteins and HA status. Herovici and Movat staining revealed neocollagenesis and neoelastogenesis, respectively, while CD44 convincingly demonstrated HA stimulation. With a special interest in fat tissue modulation, Perilipin‐1 staining was undertaken, understanding that Perilipin‐1 denotes new healthy fat cell formation [9]. Perilipin‐1 convincingly showed an increase in intact peripheral staining of new adipocyte cells at 12 weeks compared to baseline. This was demonstrated on stains and confirmed with ImageJ analysis, suggesting new fat cell modulation and replacement in the region.

These special investigations are clearly confirmed by clinical changes reported by investigators, clinical photography, and subjects themselves, as depicted by increased plumpness and radiance, decreased roughness, wrinkle assessments, and by a dramatic increase in hydration.

## 5 Conclusion

This study adopted a multifaceted approach for evaluating improvements in facial skin health through integrating 3D photography for precise visual improvement in volume and wrinkles, investigator assessments for expert clinical outcomes, patient‐reported outcomes to demonstrate subject satisfaction, and in‐depth histological evaluation to demonstrate the efficacy of this topical formulation. The convergence of this multiple modality assessment demonstrates the efficacy of Alastin Restorative Skin Complex Serum 2.0 with TriHex Technology for improving skin health and facial volume.

## Author Contributions

**Alan D. Widgerow:** developed the science, analysis, paper writing. **Charles Boyd:** study investigator, paper writing contribution. **Deanne Mraz:** study investigator, paper writing contribution. **Faiza Shafiq:** study concept, design, and supervision, data analysis, paper writing. **Michael Gold:** study investigator, paper writing contribution. **Tiffany Robison:** study design and management, data analysis, paper writing.

## Conflicts of Interest

Alan D. Widgerow (Chief Scientific Officer), Faiza Shafiq (Director Clinical Research), Tiffany Robison (Manager, Clinical Research) are all employees of Galderma.

## Data Availability

The data that support the findings of this study are available from the corresponding author upon reasonable request.
